# Grassland restoration reduces water yield in the headstream region of Yangtze River

**DOI:** 10.1038/s41598-017-02413-9

**Published:** 2017-05-19

**Authors:** Jia Li, Dan Liu, Tao Wang, Yingnian Li, Shiping Wang, Yuting Yang, Xiaoyi Wang, Hui Guo, Shushi Peng, Jinzhi Ding, Miaogen Shen, Lei Wang

**Affiliations:** 1 CAS Center for Excellence in Tibetan Plateau Earth Sciences, Chinese Academy of Sciences, Beijing, 100085 China; 2 Key Laboratory of Alpine Ecology and Biodiversity, Institute of Tibetan Plateau Research, Chinese Academy of Sciences, Beijing, 100085 China; 3 Northwest Institute of Plateau Biology, Chinese Academy of Sciences, Xining, 810001 China; 4 Key Laboratory of Adaptation and Evolution of Plateau Biota, Chinese Academy of Sciences, Xining, 810001 China; 5 CSIRO Land and Water, Canberra, Australian Capital Territory Australia; 6 Laboratoire des Sciences du Climat et de l’Environnement, Commissariat à l’Energie Atomique, Centre National de la Recherche Scientifique, Université de Versailles Saint-Quentin-en-Yvelines, 91191 Gif-sur-Yvette, France

## Abstract

Large–scale ecological restoration programs are considered as one of the key strategies to enhance ecosystem services. The Headstream region of Yangtze River (HYZR), which is claimed to be China’s Water Tower but witnessed the rapid grassland deterioration during 1970s–2000, has seen a series of grassland restoration programs since 2000. But few studies have thoroughly estimated the hydrological effect of this recent grassland restoration. Here we show that restoration significantly reduces growing-season water yield coefficient (WYC) from 0.37 ± 0.07 during 1982–1999 to 0.24 ± 0.07 during 2000–2012. Increased evapotranspiration (ET) is identified as the main driver for the observed decline in WYC. After factoring out climate change effects, vegetation restoration reduces streamflow by 9.75 ± 0.48 mm from the period 1982–1999 to the period 2000–2012, amounting to 16.4 ± 0. 80% of climatological growing-season streamflow. In contrary to water yield, restoration is conducive to soil water retention – an argument that is supported by long-term *in-situ* grazing exclusion experiment. Grassland restoration therefore improves local soil water conditions but undercuts gain in downstream water resources associated with precipitation increases.

## Introduction

**Figure 1:**
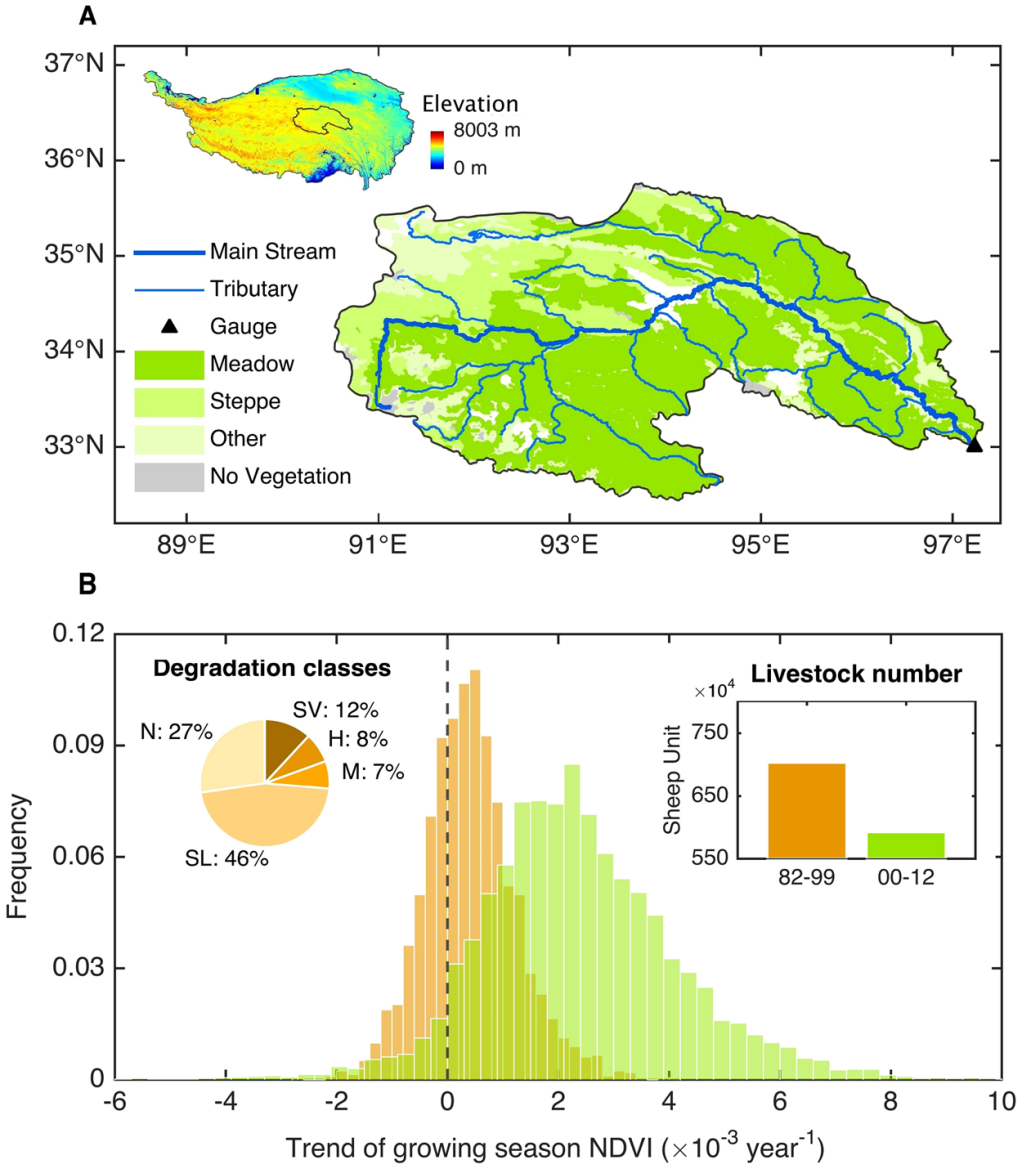
Grassland degradation and restoration in the headstream region of Yangtze River. (**A**) The spatial distribution of vegetation types in the catchment. Vegetation data is from the Institute of Botany, Chinese Academy of Sciences of 1:100 million Chinese Vegetation Map (2000). The maps in the figure is generated from MATLAB (R2014b). (**B**) Frequency distributions of growing-season (June to September) NDVI trends during pre-restoration (1982–1999) (in orange) and post-restoration (2000–2012) periods (in green). The left inset indicates the percentage for each of the five grassland degradation classes that took shape over the final three decades of the twentieth century, and the right inset shows the change in livestock number (in sheep unit) between pre-restoration and post-restoration period. Five different degradation classes are N (no degradation), SL (Slight degradation), M (Moderate degradation), H (Heavy degradation), and SV (Severe degradation), respectively. The NDVI trends during pre-restoration and post-restoration period are respectively based on GIMMS NDVI3g and MODIS, given the low data-quality of GIMMS NDVI in 2000 (Fig. S2).

The headstream region of Yangtze River on the Tibetan Plateau covers ~17% of the whole Yangtze River basin and provides nearly 20% of the water volume of the Yangtze River^1^. The headstream region holding rich alpine grasslands (Fig. 1A) witnessed severe degradation (~73% loss of original grasslands) primarily from overgrazing and rodent destruction^2, 3^ during 1970s–2000 (Fig. 1B). The increasing human-induced pressure combined with warming will potentially shift fragile grasslands to an irreversible state that no longer provides key environmental services. Since 2000, to reduce the environmental degradation, China’s state and local authorities initiate a series of rehabilitation measures such as grazing exclusion by fencing, grassland seeding and control of rodents and insects (Fig. 1B). These recent restoration efforts have yielded significant success in terms of herbage production and carbon sequestration potential^4, 5^ (see Supplementary Text S1). However, it is unclear whether the recent vegetation restoration programs have affected water resources.

Prior studies of effect of vegetation restoration on water yield focused primarily on afforestation^6–10^. Afforestation generally reduces water yield and soil moisture^8, 11^, which tends to become severe in semi-arid and arid regions that can even degrade planted trees because of aggravated soil water shortages^7^. The adoption of vegetation restoration through afforestation to combat environmental degradation in arid and semi-arid region has been questioned from water resource perspectives^7^. Have similar negative impacts on water resources also occurred after grassland restoration over the Headstream Region of Yangtze River (HYZR)? Answering this question could provide guidance for developing policies associated with ecosystem restoration over degraded semi-arid and/or arid regions.

Furthermore, there is growing evidence that vegetation can influence local precipitation by mediating moisture fluxes between land surface and the atmosphere (evapotranspiration)^12–14^, thereby contributing to changes in water yield (precipitation minus evapotranspiration and water storage change). Evidence from isotopic compositions of precipitation showed that Tibetan Plateau is featured by relatively high moisture recycling^2, 15, 16^. Therefore, assessing the impact of vegetation restoration on water yield through local vegetation-precipitation feedback has significant implications for the headstream region of the Yangtze River. However, previous assessments of ecosystem restoration on water yield barely quantify the contribution from vegetation change-induced precipitation change.

In this study, we evaluate the impact of recent grassland restoration on water resources in the headstream of Yangtze River based on the Budyko framework and an offline atmospheric moisture tracking model, with the simultaneous use of streamflow observations, a model-data fusion product blending process-based understanding with multi-satellite observations of climatic and environmental variables^17^, and *in*-*situ* long-term grazing exclusion experiments.

## Results

### Observed decline of water yield coefficient at the catchment scale

**Figure 2:**
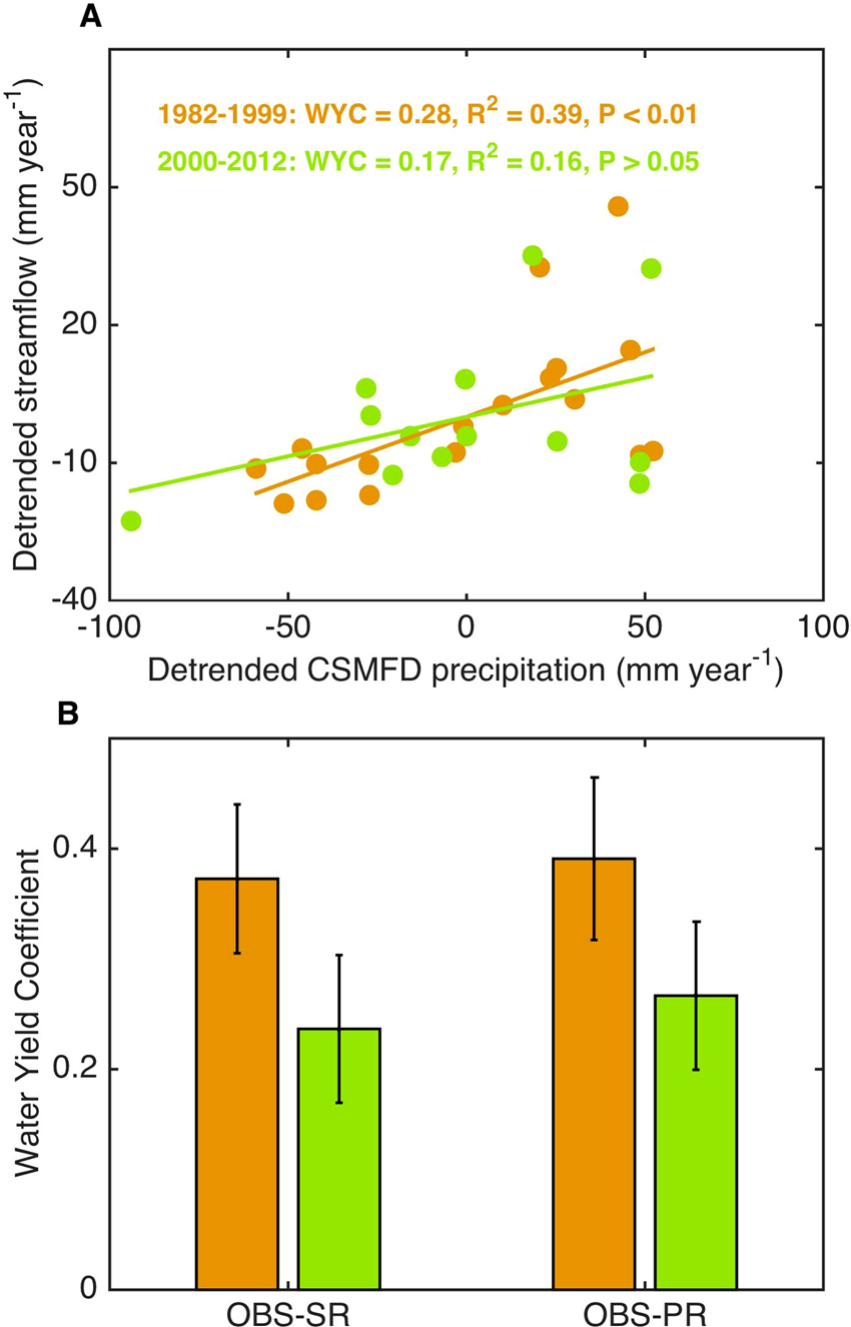
The relationship between precipitation and streamflow at Zhimenda hydrological station. (**A**) Relationship of streamflow with precipitation from China Surface Meteorological Forcing Dataset (CSMFD) (all variables detrended); (**B**) Water yield coefficient (WYC) for the pre-restoration and post-restoration period using the three different precipitation products (MSWEP, CSMFD, and CRU). The abbreviations for each precipitation product can be referenced to Datasets. **OBS-SR** denotes the slope calculated between streamflow and precipitation (all variables detrended), and **OBS-PR** is the partial derivative of streamflow with respect to precipitation in a multiple regression of streamflow against precipitation and CSMFD temperature (all variables detrended).

The linear regression slope between yearly growing-season (June-September) streamflow and yearly growing-season precipitation denotes water yield coefficient (WYC). Using precipitation data from China Surface Meteorological Forcing Dataset (CSMFD) and streamflow data from Zhimenda hydrological station at the outlet of HYZR, WYC decreases from 0.28 (*p* < 0.01) during 1982–1999 to 0.17 (*p* > 0.05) during 2000–2012 (Fig. 2A) (all variables detrended, and the difference of WYC between the two periods passed significance test at *p* < 0.05). To test if the decline of WYC could depend on the choice of a climate forcing dataset, we used different precipitation products (Fig. 2B). The results based on other precipitation products confirm that the decrease of WYC after the implementation of restoration project is a robust finding (see Supplementary Fig. S3A). The WYC from all precipitation products decreases from 0.37 ± 0.07 during 1982–1999 to 0.24 ± 0.07 during 2000–2012 (Fig. 2B). When interpreting this observed decrease of WYC, it might be argued that it does not reflect the change in the true response of streamflow to precipitation variations, but could be due to indirect effects of temperature. We therefore calculate the WYC as the partial derivative of streamflow with respect to precipitation in a multiple regression of streamflow against precipitation and CSMFD temperature. The decline in WYC is also found if the effect of temperature on streamflow was removed (Fig. 2B). Similar results are also obtained if all variables were not detrended (see Supplementary Figs S4 and S5).

### Quantifying the impact of grassland restoration on growing-season water yield change

We show that the WYC declines during the post-restoration period, but the streamflow at the outlet of HYZR do exhibit an increase of 14.6 mm during the post-restoration period relative to the pre-restoration period. This seemingly paradox arises from a corresponding increase of catchment-scale precipitation (73.5 mm) based on MSWEP during the latter period. The quantification of grassland regeneration on water yield change is therefore complicated by accompanied climate change.

**Figure 3:**
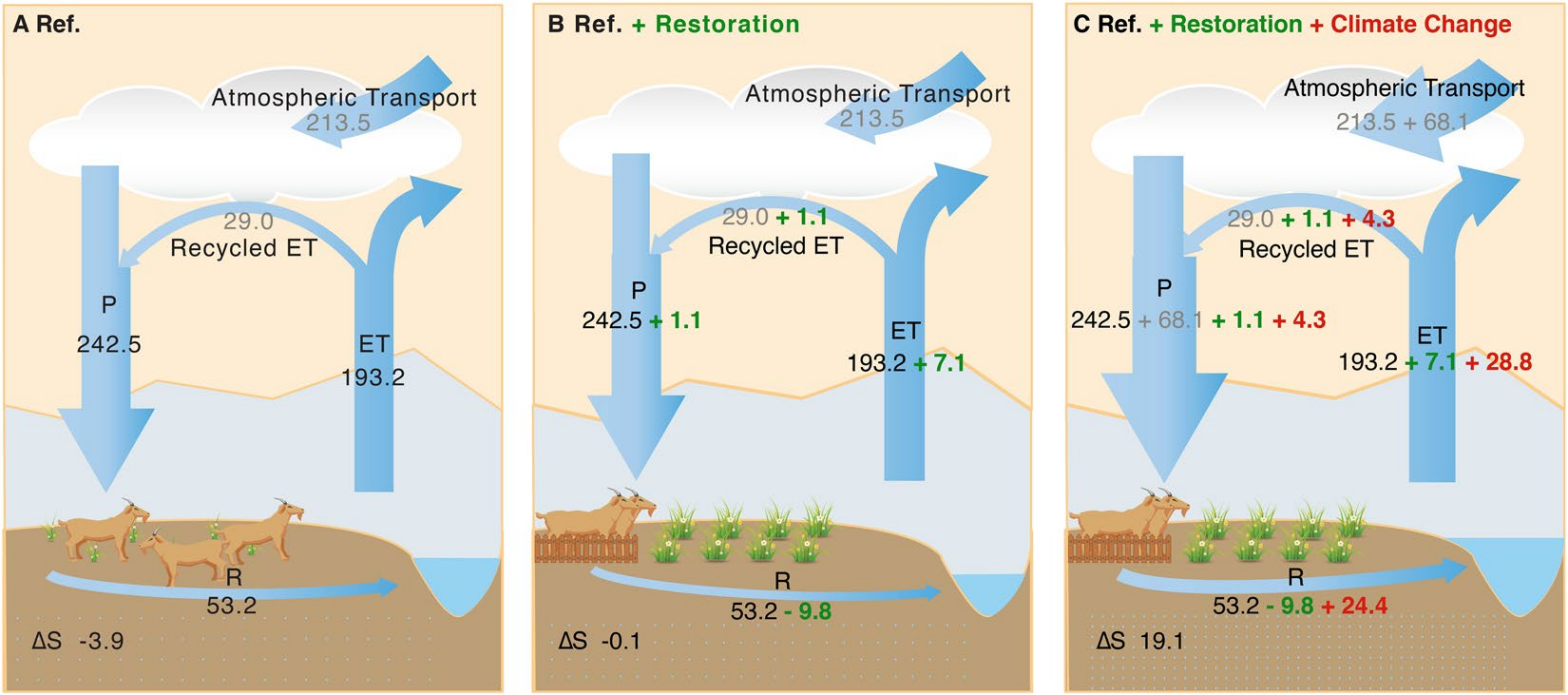
Changes in catchment water balance components in response to grassland restoration and climate change in the headstream region of the Yangtze River. (**A–C**) Represent three different scenarios for water balance components during the growing season (June-September). (**A,C**) Are “true” scenarios that denote the mean state of water balance components during pre-restoration (reference period) and post-restoration period, respectively. (**B**) Represents a pseudo-scenario with restored grassland but no climate change relative to the reference period. Precipitation (P) and evapotranspiration (ET) are derived from MSWEP and GLEAM respectively. R is the observed streamflow from Zhimenda hydrological station situated at the outlet of HYZR. ΔS represents change in root-zone soil water storage that is calculated as the residual based on catchment water balance equation (R = P − ET − ΔS). The numbers in black denote the data taken from either observations or products. The numbers in gray indicate that these values are estimated based upon atmospheric moisture tracking model. The numbers in green and red represent changes in water balance components due to grassland restoration and climate change respectively, and the methods used to estimate these numbers are referenced to the main text (see Data and Methods). Note that Ref. denotes the reference period or the pre-restoration period. This image is generated from Adobe Illustrator CC software.

Figure 3 summarizes a quantitative assessment of changes in streamflow and its main driver related to grassland restoration and climate change between pre-restoration and post-restoration period. First, we apply a sensitivity-based approach (see Data and Methods) to separate contributions of climate change and grassland regeneration to streamflow change between the two time periods. There is a decrease of 9.75 ± 0.48 mm in streamflow due to grassland regeneration (Fig. 3B), and the magnitude of the decrease can amount to 16.4 ± 0. 80% of mean streamflow (59.3 mm). This decline is, however, over-compensated by an increase of 24.4 mm due to climate change therefore resulting in an observed increase of 14.6 mm in streamflow (Fig. 3C).

Second, we examine the relative contribution of changes in ET (actual evapotranspiration) and soil moisture to streamflow changes after vegetation restoration, using the diagnostic dataset GLEAM^17^ in which ET (mm) and root-zone soil moisture (SM, m^3^ m^−3^) are modeled by assimilating satellite observations (see Section 2.1). By removing the impact of climate change on total ET change according to the Budyko framework (see Data and Methods), we estimate an increase of ET due to grassland regeneration (7.1 mm, Fig. 3B; in contrast with a total ET increase of 35.9 mm, Fig. 3C), the magnitude of which is comparable to restoration-induced reduction in streamflow (9.75 ± 0.48 mm). This result suggests that increased ET is mainly responsible for the decline in streamflow due to grassland restoration.

Besides increased ET, water storage, which is computed as the residual based upon the catchment water balance equation, also increases following grassland restoration (Fig. 3B). To gain further mechanistic insights, we use local grazing exclusion experiments performed at two typical alpine grasslands (steppe and meadow) on Qinghai-Tibetan Plateau to understand the effect of long-term fencing (a widely-used approach for restoring degraded grasslands). In response to grazing exclusion lasting for more than 10 years, standing aboveground biomass has an increase of 30.5% and 70.5% in alpine steppe and meadow, respectively (Table 1), demonstrating the effectiveness of long-term fencing in restoring degraded grassland. We find that the total soil water storage in the top 40 cm is higher in fencing plots (steppe: 26.7 mm and meadow: 109.2 mm) than that in grazing plots (steppe: 25.4 and meadow: 95.0 mm) (Table 1). The enhanced soil water retention is mainly as a result of elevated maximum soil water holding capacity after long-term fencing, with an increase of 15.3% and 9.9% in alpine steppe and meadow respectively (Table 1). This enhanced capacity is intrinsically linked with decreased bulk density due to the beneficial effects of removed herbivore trampling and root development. *In-situ* grazing exclusion experiment provides support for an increase in catchment*-*scale root-zone soil moisture, and this observed increase in soil water could further sustain plant transpiration that is often constrained by soil moisture availability in the semi*-*arid and arid region.

**Table 1:** Summary of long-term fencing effect on ecosystem characteristics on two alpine grasslands.

Variables	Alpine steppe		Alpine meadow	
	Fencing	Grazing	Fencing	Grazing
Soil water content (mm)	26.7	25.4	109.2	95.0
Bulk density (g cm^−3^)	1.65	1.75	0.92	1.05
MSWHC (mm)	39.2	34.0	60.0	54.6
Aboveground biomass (g m^−2^)	54.7	41.9	123.1	72.2

Shown are soil properties: soil water content in the top 40 cm, bulk density, and Maximum Soil Water Holding Capacity (MSWHC); and aboveground biomass on the two typical alpine grasslands (steppe and meadow).

Third, we further examine how much of the enhanced evapotranspiration due to grassland restoration contributes to changes in streamflow through regional evaporation recycling (ε_r_). To estimate this contribution, we first compute regional ε_r_ in HYZR based on the offline Eulerian atmospheric moisture tracking model (Water Accounting Model-2layers, WAM-2layers)^18^ and find that about ~15.2% of the evaporation returns as precipitation over HYZR. Combining the computed ε_r_ (~15.2%) with the estimated ET increase due to grassland regeneration (7.10 mm) yields an increase of 1.10 mm due to regional evaporation recycling. According to the observed streamflow-precipitation ratio (0.20) during 2000–2012, this precipitation increase can translate into an increase of 0.22 mm in water yield due to vegetation regeneration. Our results indicate that the increase in streamflow due to regional evaporation recycling is rather small (0.22 mm), which cannot meaningfully compensate for the water loss through enhanced ET.

## Discussion

This study is a first attempt to evaluate water supply benefits from catchment-scale grassland restoration in HYZR. Our catchment-level results show that there is a loss in growing-season flow in the restored grassland, mainly due to increased ET and enhanced soil water retention. The ecosystem service value from restoration project may be less than assumed from the perspective of generating more waters in the river. Nevertheless, it is important to emphasize that such restoration actions are valuable for soil and water conservation for the headwater catchment ecosystems since it improves the soil hydrological function (bulk density and maximum soil water holding capacity)^19^. This finding contrasts with previous studies focusing on the assessment of the hydrological effects of extensive afforestation in arid and semi-arid region^7, 11^, which could extract more underground water and result in severe soil moisture depletion due to a high water demand.

We show that the increase of water yield induced by grassland restoration through regional evaporation cycling is rather small. However, this estimate might be conservative since restoration-induced changes in precipitation due to atmospheric dynamic changes are not considered in WAM-2layers moisture tracking model. We cannot rule out the possibility that grassland restoration implemented in a much broader spatial scale (also occurring outside of the headstream region of Yangtze River) would develop (or intensify) low atmospheric pressure systems due to enhanced evapotranspiration, therefore resulting in a strengthening of moisture convergence (increased precipitation). In the future study, resorting to a coupled modeling strategy by considering the interactions among land, atmosphere and humans is therefore necessary to fully evaluate the impact of grassland restoration project on the hydrological cycle.

Finally, we should inform that HYZR holds glaciers and a large extent of permafrost, covering ~0.95% and ~75% of the total area respectively. In the past three decades, air temperature increases at a rate of 0.04 °C year^−1^ over HYZR, which contributes to melting of glaciers^20^ and triggers permafrost degradation^21^. Although root-zone soil moisture provided by GLEAM is modeled through assimilating microwave surface soil moisture (0–0.05 m) in three-layer soil water budget, it cannot fully account for changes in total soil moisture attributable to permafrost degradation and glacier melting. Glacier retreat could release frozen water and potentially contributes to the increase in water yield. It therefore might not be the main reason for the observed decline in WYC. In addition, the effect of permafrost degradation on water yield is complex. On the one hand, the increase of active layer thickness due to permafrost degradation would increase soil water storage capacity, which would lead to the reduction of water yield. On the other hand, the release of frozen water in the ice-rich permafrost might compensate for the negative effect due to increasing water holding capacity in the soils. Therefore the contributing effect of permafrost degradation to the observed WYC decline (if exists) would not be expected too large. But it is uncertain in the future characteristic with a more pronounced warming over HYZR, since which distributes a large portion of permafrost with a relatively high temperature^21^. We therefore suggest that impact of permafrost changes on hydrological cycle requires urgent attention, including how characteristics of permafrost feedback to the water cycle (such as ground ice content and effect of changes in active layer thickness on infiltration and soil water-holding capacity).

In summary, such reduced streamflow responses after grassland restoration should be considered in future projections of downstream water. Continued pursuant of ecological restoration will reduce gain in downstream water resources due to precipitation increases. But this project can contribute to summer flood control since future climate change is likely to cause more frequent extreme floods in the Yangtze basin^22^. Moreover, improved soil retention after the implementation of restoration project should create benefits for reducing sediment transport in the Yangtze River that ranks fourth in sediment flux globally^23^.

## Data and Methods

### Datasets

The precipitation dataset during the period 1982–2012 is taken from China Surface Meteorological Forcing Dataset (CSMFD) (3-hourly, 0.1° × 0.1° grid) that merges observations of meteorological stations with the model reanalysis^24^. More stations are used in CSMFD than other available datasets. In addition, we also used precipitation dataset from Multi-Source Weighted-Ensemble Precipitation (MSWEP) with a 3-hourly temporal and 0.25° spatial resolution^25^, and the Climatic Research Unit (CRU, monthly, 0.5° × 0.5° grid)^26^. MSWEP precipitation data optimally merge different high-quality precipitation data sources (interpolation of gauge observations, satellite remote sensing and atmospheric model reanalysis). For each grid cell, the weight for gauge-based estimates is based on the gauge network density, and the weights for satellite- and reanalysis-based estimates are computed from their performance at the surrounding gauges^25^. Daily streamflow data are obtained from the gauged hydrological station (Zhimenda) situated at the outlet of HYZR, and the river flow regimes in HYZR are much less affected by human intervention such as dam construction and water withdrawals than the middle and lower reaches of Yangtze River.

Satellite-derived Normalized Difference Vegetation Index (NDVI) as a surrogate of biomass and vegetative cover changes^27, 28^ is adopted to assess the effectiveness of ecological protection and restoration projects implemented in the study region. We use biweekly NDVI data of 1982–2012 from the third-generation GIMMS (Global Inventory Modeling and Mapping Studies), with a spatial resolution of 0.083°. In addition, we also use NDVI from the National Aeronautics and Space Administration Earth Observing System’s satellite Terra (MODIS) (500 m and 16d) during 2000–2012. All NDVI are aggregated to 0.5° × 0.5° to match the resolution of meteorological data.

Potential, actual evapotranspiration and root-zone soil moisture, with spatial and temporal resolutions of 0.25 degrees and 1 day, are extracted from the version 3.0a dataset of Global Land Evaporation Amsterdam Model (GLEAM)^17, 29^. The GLEAM estimates terrestrial evaporation based on daily satellite observations of meteorological drivers of terrestrial evaporation, vegetation characteristics and soil moisture. The forcing variables used in generating GLEAM product are based on air temperature and radiation from the European Centre for Medium-Range Weather Forecasts Interim Re-Analysis (ERA-interim), MSWEP precipitation, surface soil moisture from The European Space Agency (ESA)-Climate Change Initiative (ESA-CCI), vegetation optical depth from Land Parameter Retrieval Model (LPRM) and snow water equivalent from Global Snow Monitoring for Climate Research (GlobSnow). The Priestley and Taylor equation is used to calculate potential evapotranspiration and actual evaporation is then converted by the evaporative stress factor that is computed based on vegetation optical depth (as a proxy of vegetation water content) and simulations of root-zone soil moisture. A multi-layer water balance module, which describes the infiltration rates as function of the vertical gradient in soil moisture, is used to simulate root-zone soil moisture by assimilating microwave observations of surface soil moisture from ESA-CCI dataset. Even though glacier melting is not included as an input, the assimilation of the satellite soil moisture in GLEAM could partly account for it by adjusting the soil moisture seasonal dynamics of the area. The MSWEP precipitation and GLEAM product can be downloaded from the website (http://www.gleam.eu/). GIMMS NDVI is available at https://ecocast.arc.nasa.gov/data/pub/gimms/3g.v1/.

### Grassland degradation and restoration in the studied region

The headstream region of the Yangtze River (HYZR) ranges from 90°43′ to 97°31′E longitude and 32°30′–35°35′N latitude with an area of 1.28 × 10^5^ km^2^ (Fig. 1A), accounting for 43.2% of Three-River (Yangtze, Yellow and Mekong) Headwaters region. The main grassland type in HYZR is alpine meadow and alpine steppe, which was degraded to form “black–soil–patch” grassland and desertification respectively. Irrational overstocking of livestock and rodent destruction has been widely cited as the principle culprit for grassland degradation^2, 3^. Interpretation of satellite imageries showed that the pattern of grassland degradation in HYZR took shape initially in the mid and late 1970s^2^.

In 2000, the government of Qinghai Province established the Three-River Headwaters reserve and this aroused great concerns of the Chinese central government. Thus in 2003, the State Council officially approved creating the Three Rivers Headwaters Nature Reserve. In 2005, the Chinese government has allocated a spending over $US 1.1 billion on “The project of ecological protection and construction for the Three-River Headwaters Region nature reserve” program, which is the largest project for nature reserve protection and reconstruction in China. Since then, the widespread application of the reduction of livestock project (Fig. 1B) and artificial management of “black–soil–patch” through planting grasses, killing weeds and fertilization^3^ have been carried out for restoring seriously degraded grassland ecosystems.

### Sensitivity-based approach to separate effects of grassland restoration on streamflow

The total streamflow change between post-restoration and pre-restoration period can be partitioned into the streamflow change due to climate change and that due to changes in catchment characteristics^30, 31^. In this study, change in catchment characteristics can be mainly attributed to vegetation regeneration since the headstream of Yangtze River is not significantly affected by human intervention such as dam construction and water withdrawals.

1 $${\rm{\Delta }}{Q}_{tot}={\rm{\Delta }}{Q}_{veg}+{\rm{\Delta }}{Q}_{clim}$$

we estimate Δ*Q*_*clim*_ from Δ*P* and Δ*ET*_*P*_ (potential evapotranspiration change) between post-restoration and pre-restoration period based on the following equations^31–33^,

2 $${\rm{\Delta }}{Q}_{clim}=({\varepsilon }_{P}\frac{{\rm{\Delta }}P}{\bar{P}}+{\varepsilon }_{EP}\frac{{\rm{\Delta }}E{T}_{P}}{\overline{E{T}_{P}}})\bar{Q}$$

3 $${\varepsilon }_{P}+{\varepsilon }_{E{T}_{P}}=1$$

4 $${\varepsilon }_{P}=1+\frac{AIf^{\prime} (AI)\,}{1-f^{\prime} (AI)}$$

5 $$AI=\frac{\overline{E{T}_{P}}}{\bar{P}}$$

6 $${\rm{f}}(AI)=1-\exp (\,-\,{\rm{AI}})$$

7 $$f^{\prime} (AI)=\exp (\,-\,{\rm{AI}})$$

where *ε*_*P*_ and ${\varepsilon }_{E{T}_{P}}$ are sensitivity coefficient of streamflow to precipitation and potential evapotranspiration, respectively. $\bar{P}$, $\overline{E{T}_{P}}$ and $\bar{Q}$ represent long-term (1982–2012) mean of effective precipitation, potential evapotranspiration and streamflow during the growing season. *AI* is aridity index. In addition, we also use other five commonly used forms of f(*AI*) and *f* ′(*AI*) in calculating *ε*_*P*_ (see Supplementary Table S1). Both root-zone soil moisture and potential ET are directly taken from the GLEAM product.

### The Budyko framework to estimate climate-driven evapotranspiration

Total evapotranspiration change (Δ*ET*_*tot*_) can be separated into Δ*ET*_*tot*_ = Δ*ET*_*clim*_ + Δ*ET*_*veg*_, where Δ*ET*_*clim*_ is the contribution from climate change and Δ*ET*_*veg*_ is the remaining change contribution from grassland restoration. We estimate climate-driven ET change following the Budyko equation of the generalized form^34^,

8 $$ET=P{[1+{(\frac{E{T}_{p}}{P})}^{-\omega }]}^{-\frac{1}{\omega }}$$

where *ET*, *ET*_*P*_ and *P* represent actual evapotranspiration, potential ET and precipitation during the growing season, respectively, and *ω* denotes catchment characteristics.

We first fit Eq. (8) using the GLEAM data during the pre-restoration period (1982–1999) to obtain the parameter *ω* based on non-linear iterative Nelder-Mead simplex optimization algorithm^35^. To evaluate the uncertainty of parameter *ω*, we randomly selected 12 year data from the period 1982–1999 to fit the Eq. (8) for 5000 times, and generated the frequency distribution of *ω* (Fig. S6). We obtained the mean value of *ω* for further analysis. The parameter *ω*, which is assumed to be constant during the post-restoration period (2000–2012) (that is, no change in catchment characteristics), is then used to estimate climate-driven ET following Eq. (8). The difference between actual ET from GLEAM and the estimated climate-driven ET during the period 2000–2012 can therefore be referenced to the residual ET due to vegetation regeneration. We should note that other factors such as climate seasonality^36^ also impact the parameter *ω*, which is simply assumed to be only related to catchment characteristics in this study.

### Grazing exclusion experiments

We have established livestock manipulation experiments to investigate the impact of grazing exclusion on ecosystem soil water retention at both alpine steppe (34°55′N, 98°10′E, 4270 m) and meadow (34°28′N, 100°12′E, 3763 m) on Tibetan Plateau. Alpine steppe and meadow are respectively dominated by *Stipa apurpurea* and *Kobresiamyosuroides* (Villars) Foiri. At both sites, the control (or grazing) plot is established 500 m away from the fencing plot with a complete exclusion of livestock grazing from June to October since 2003. The grazing intensity was 1.36 sheep per hm^2^ and the sizes of fencing and grazing plots are 60 m × 60 m.

In 2013, *in-situ* soil moisture content at different soil depths (every 10 cm in the 0–40 cm soil profile) with three replicates is measured monthly from June to September (8th, 18th and 28th day in each month) in both fencing and grazing sites. At the end of August in 2013, soil samples at depth intervals of 0–10, 10–20, 20–40 cm were collected using a soil wreath knife with 100 cm^3^ in volume, and then oven-dried at 105 °C for determining bulk density. In order to determine maximum water holding capacity, we cover the bottom of the soil tube stored in the soil wreath knife with a wet piece of filter paper and then place it in a water bath. The soil tube is submerged until the water level is above to the top of the soil and is then left in the water for about 12 hours. The soil sample is then oven-dried 105 °C to quantify maximum water holding capacity. In addition, the total soil water storage over the depth of 40 cm is also calculated by considering the bulk density, soil layer depth and soil moisture content at different depths.

### The impact of grassland restoration on water yield due to local moisture recycling

We use offline atmospheric moisture tracking model (Water Accounting Model – two layers, WAM-2layers)^18^ to compute regional evaporation recycling (the fraction of terrestrial evapotranspiration returns as rainfall within the same region) in the headstream region of the Yangtze River. Compared to complex moisture tracking scheme in a regional climate model^37^, WAM-2layers has very similar results with much smaller computational cost^18^. The input data is taken from the ERA-Interim reanalysis (ERA-I) on a 1.5° latitude × 1.5° longitude grid for the period of 1982–2012^38^. We use multi-year mean growing season (June to September) recycling ratio during the period 2000–2012 (15.2%) to compute the amount of precipitation change following changes in evaporation due to grassland restoration. The mean observed ratio of streamflow at the outlet of HYZR to catchment-scale precipitation during the growing season for the period 2000–2012 is then adopted to estimate water yield change from restoration-induced precipitation change due to regional evaporation recycling.

### Analyses

The water yield-precipitation relationship is the viable option that can be adopted to diagnose how changes in catchment characteristics such as vegetative cover affects the proportion of precipitation running off a given catchment area. In the analysis, we use the slope (or sensitivity) of the streamflow/precipitation relationship (hereafter water yield coefficient, see below) at the catchment scale to evaluate the effect of grassland restoration in HYZR.

The study period is confined to the period 1982–2012, which is based on the availability of gauzed Zhimenda streamflow data, satellite observations and meteorological data sets. The growing season is defined as June-September. The water yield coefficient is calculated as the slope between growing-season precipitation and streamflow in both pre-restoration (1982–1999) and post-restoration periods (2000–2012). Using the similar method, we also calculate the water yield coefficient based on CRU precipitation and modeled streamflow from runoff simulations taken from terrestrial biosphere models. In addition, to evaluate the effectiveness of grassland restoration project in NDVI, we analyze NDVI linear trends derived from the least squares method. The trend analyses are first performed for the entire HYZR and then for each pixel.

## Electronic supplementary material

Supplementary Info
