# Supplementary material for: Grassland restoration reduces water yield in the headstream region of Yangtze River
Source: Sci Rep. 2017 May 19;7:2162. doi: 10.1038/s41598-017-02413-9 (PMC5438355; doi:10.1038/s41598-017-02413-9)
Supplement: Supplementary file 1 — Supplementary Info [file 41598_2017_2413_MOESM1_ESM.pdf]

Supplementary Information for

Grassland restoration reduced water yield in the headstream region of Yangtze River

Jia Li[1,2,§], Dan Liu[2,§], Tao Wang[1,2], Yingnian Li[3,4], Shiping Wang[1,2], Yuting Yang[5], Xiaoyi Wang[2], Hui Guo[2], Shushi Peng[6], Jinzhi Ding[2], Miaogen Shen[1,2], Lei Wang[1,2]

[1]CAS Center for Excellence in Tibetan Plateau Earth Sciences, Chinese Academy of Sciences, Beijing 100085, China

[2]Key Laboratory of Alpine Ecology and Biodiversity, Institute of Tibetan Plateau Research, Chinese Academy of Sciences, Beijing 100085, China

[3]Northwest Institute of Plateau Biology, Chinese Academy of Sciences, Xining 810001, China

[4]Key Laboratory of Adaptation and Evolution of Plateau Biota, Chinese Academy of Sciences, Xining 810001, China

[5]CSIRO Land and Water, Canberra, Australian Capital Territory, Australia

[6]Laboratoire des Sciences du Climat et de l'Environnement, Commissariat à l'Energie Atomique, Centre National de la Recherche Scientifique, Université de Versailles Saint-Quentin-en-Yvelines, 91191 Gif-sur-Yvette, France

§: Both authors contributed equally to this work.

Address correspondence to:

Dr. Tao Wang,

Institute of Tibetan Plateau, Chinese Academy of Sciences, Beijing, China

Email: twang@itpcas.ac.cn

Dr. Yinnian Li,

Northwest Institute of Plateau Biology, Chinese Academy of Sciences, Xining, China

Email: ynli@nwipb.cas.cn

Contents of this file

Text S1, Table S1, Figures S1 to S6

Text S1. Grassland regeneration after implementation of restoration projects

Since 2000, we do observe an increasing trend in basin-wide NDVI (being an indicator of vegetative cover) (Figs S1 and S2). Basin-wide NDVI increases at a rate of 0.012 decade^{-1} since 2000 with no significant change ($P > 0.05$), While this increase becomes statistically significant (0.025 decade^{-1}, $P < 0.05$) if the year 2000 was removed. We exclude the year 2000 given that GIMMS might have severe data quality issues in most parts of the western Plateau[1], which is supported by comparing GIMMS NDVI with the MODIS NDVI product (Fig S1).

The spatial distributions of the growing-season (GS) NDVI linear trends for 1982-1999 and for 2000-2012 are also analyzed. For 1982-1999, the GS NDVI trend map shows decrease in 30.8% of pixels (of which 2.9% are significantly negative, $P < 0.05$). In contrast, during the period from 2000 to 2012 (excluding 2000), the GS NDVI trend was positive in 95.2% of pixels (of which 47.8% are significantly positive, $P < 0.05$). Our results at both local and regional scales have shown a widespread re-greening during the latest period (2000-2012), tentatively suggesting the effectiveness of relieved grazing pressure in regenerating degraded grassland.

Expression form	$f(AI)$	$f'(AI)$	Q_{veg} (mm)
Schreiber[1]	$1 - e^{-AI}$	e^{-AI}	-10.49
Ol'dekop[2]	$AI\ tanh(1/ai)$	$tanh(1/AI) - 4/[AI(e^{-1/AI} + e^{1/AI})^2]$	-9.45
Budyko[3]	$AI\ tanh(1/AI)(1 - e^{-AI})^{0.5}$	$0.5[AI\ tanh(1/AI)(1 - e^{-AI})]^{-0.5}[(tanh(1/AI) - 1/AI\ sech^2(1/AI))(1 - e^{-AI}) + AI\ tanh(1/AI)e^{-AI}]$	-10.06
Pike[4]	$1/\sqrt{1 + AI^{-2}}$	$1/[AI^3(1 + (1/AI)^2)^{1.5}]$	9.91
Fu[5]	$1 + AI - (1 + AI^{\alpha})^{1/\alpha}, \alpha = 2.5$	$1 - (1 + AI^{2.5})^{-0.6}AI^{1.5}$	-9.42
Zhang[6]	$(1 + \omega AI)/(1 + \omega AI + 1/AI), \omega = 1$	$(2/AI + 1/AI^2)/(1 + AI + 1/AI)^2$	-9.19

Table S1. Summary of commonly-used expressions for the Budyko framework. The six commonly used forms of expressions, their first derivatives based on the Budyko framework, and the computed restoration-induced changes in streamflow (Q_{veg})

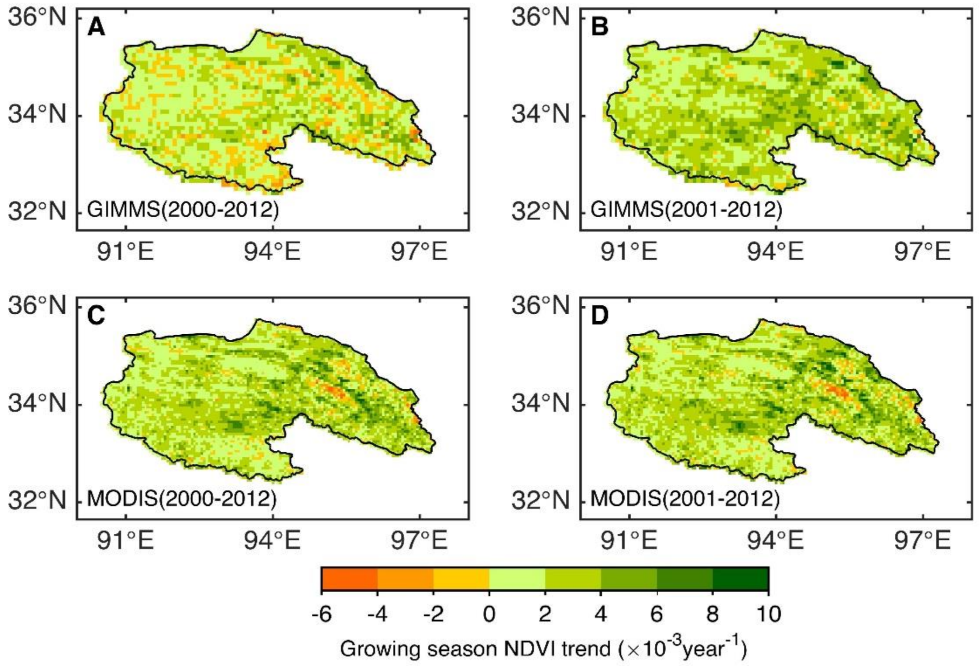

Figure S1. Spatial distribution of growing-season NDVI trends using the two datasets. A GIMMS (2000-2012); **B.** GIMMS (2001–2012); **C.** MODIS (2000-2012); **D.** MODIS (2001–2012). The maps are generated from MATLAB (R2014b).

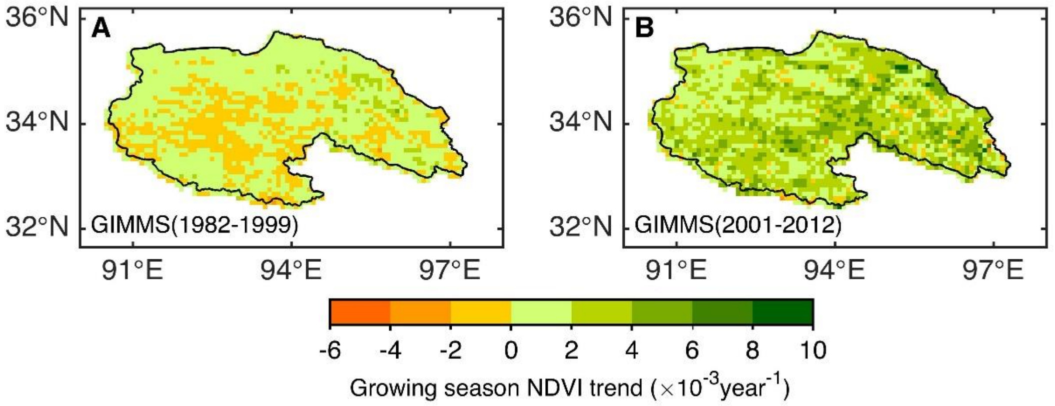

Figure S2. Spatial distribution of growing-season NDVI trends during 1982-1999 and 2001-2012 based on GIMMS. The maps are generated from MATLAB (R2014b).

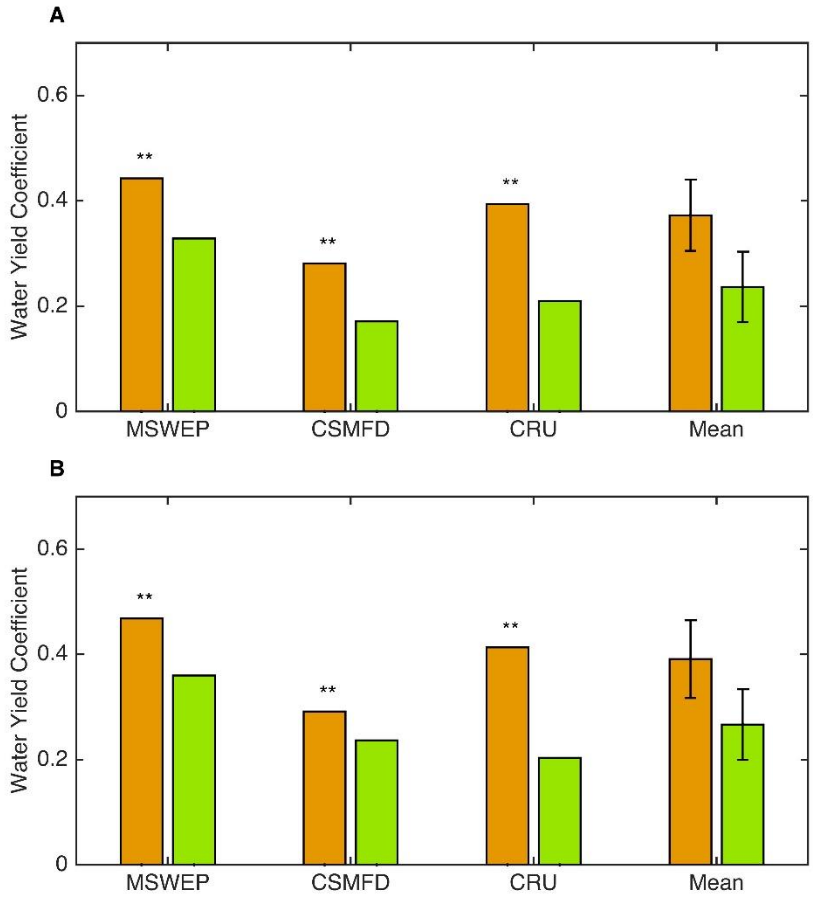

Figure S3. The water yield coefficient (WYC) during pre-restoration (1982-1999) and post-restoration period (2000-2012) for different precipitation products. A. WYC calculated as a slope between streamflow and precipitation for each precipitation product (MSWEP, CSMFD and CRU). The abbreviations for each precipitation product can be referenced to Datasets. **B.** WYC is calculated as the partial derivative of streamflow with respect to precipitation in a multiple regression of streamflow against precipitation and CSMFD temperature. All variables are detrended.

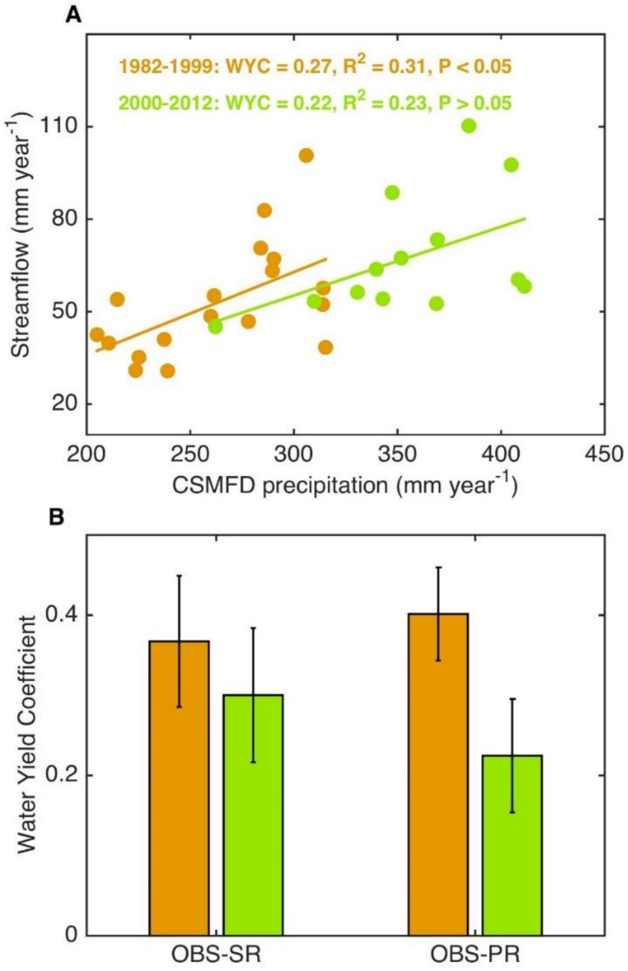

Figure S4. The relationship between precipitation and streamflow at Zhimenda hydrological station. **A.** Relationship of streamflow with precipitation from China Surface Meteorological Forcing Dataset (CSMFD); **B.** Water yield coefficient (WYC) for the pre-restoration and post-restoration period using the three different precipitation products (MSWEP, CSMFD and CRU). Note that post-restoration period is defined as 2000-2010 for the models. The abbreviations for each precipitation product can be referenced to Datasets. **OBS-SR** denotes the slope calculated between streamflow and precipitation, and **OBS-PR** is the partial derivative of streamflow with respect to precipitation in a multiple regression of streamflow against precipitation and CSMFD temperature. All variables are non-detrended.

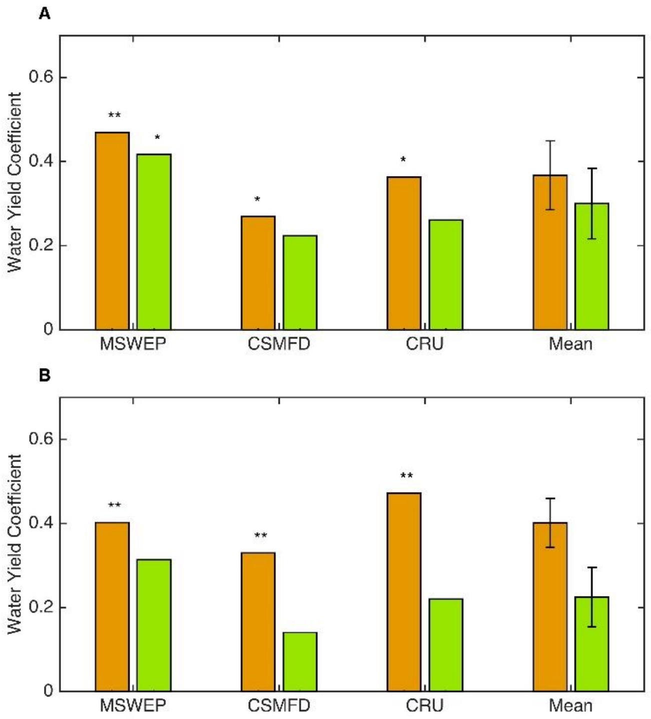

Figure S5. The water yield coefficient (WYC) during pre-restoration and post-restoration period for different precipitation products. A. WYC calculated as a slope between streamflow and precipitation for each precipitation product (MSWEP, CSMFD and CRU). The abbreviations for each precipitation product can be referenced to Datasets. **B.** WYC calculated as the partial derivative of streamflow with respect to precipitation in a multiple regression of streamflow against precipitation and CSMFD temperature. All variables are non-detrended.

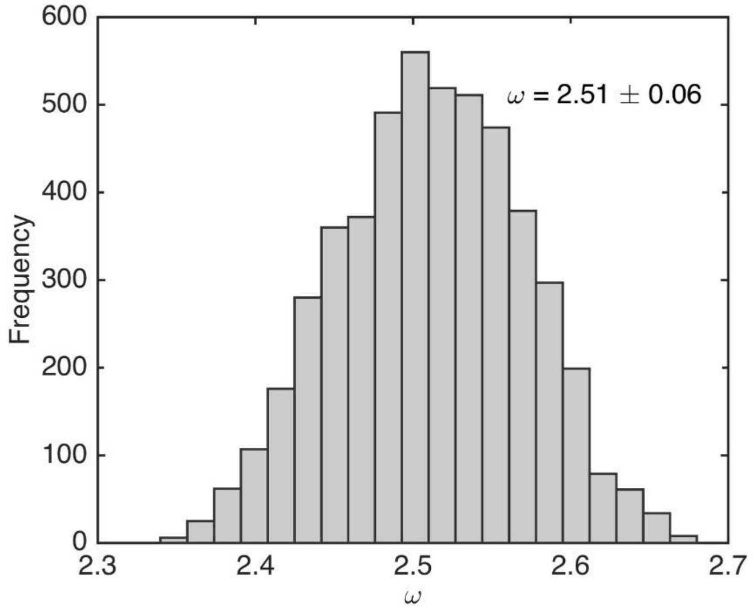

Figure S6. The frequency distribution of parameter $\boldsymbol{\omega}$ **in the Budyko framework.** The uncertainty of parameter ω is estimated by randomly selecting 12 year data from the period 1982-1999 to fit the Budyko equation for 5000 times.

References

1. Schreiber, P. Über die Beziehungen zwischen dem Niederschlag und der Wasserführung der Flüsse in Mitteleuropa. *Z. Meteorol* **21**, 441-452 (1904).
2. Ol'Dekop, E. Ob isparenii s poverkhnosti rechnykh basseinov (On evaporation from the surface of river basins). *Trans. Meteorol. Observ. Lur-evskogo, Univ. Tartu* **4** (1911).
3. Budyko, M. I. The heat balance of the earth's surface. *Soviet Geography* **2**, 3-13 (1961).
4. Pike, J. The estimation of annual run-off from meteorological data in a tropical climate. *J. Hydrol.* **2**, 116-123 (1964).
5. Fu, B. On the calculation of the evaporation from land surface. *Sci. Atmos. Sin.* **5**, 23-31 (1981).
6. Zhang, L., Dawes, W. R. & Walker, G. R. Response of mean annual evapotranspiration to vegetation changes at catchment scale. *Water Resour. Res.* **37**, 701-708 (2001).
